# Pilot Study of a Cognitive Behavioral Therapy Protocol via Videoconference for the Management of Pain, Emotional Distress, and Quality of Life in Mexican Patients with Cancer and Chronic Pain

**DOI:** 10.1007/s10880-025-10120-1

**Published:** 2026-01-07

**Authors:** Luis Alberto Mendoza-Contreras, María del Rocío Guillén-Núñez, David Alberto Rodríguez-Medina, José Fernando Mora-Romo, Oscar Galindo-Vázquez, Benjamín Domínguez-Trejo

**Affiliations:** 1 Facultad de Psicología, National Autonomous University of Mexico, Mexico City, Mexico; 2 Clínica del Dolor, Instituto Nacional de Cancerología, Tlalpan, Mexico; 3 Académica de Gerontología, Instituto de Ciencias de la Salud, Universidad Autónoma del Estado de Hidalgo, Hidalgo, Mexico; 4 Unidad Académica de Psicología, Autonomous University of Zacatecas, Zacatecas City, Mexico; 5 Unidad de Oncología Integrativa, Instituto Nacional de Cancerología, Tlalpan, Mexico

**Keywords:** Cognitive Behavioral Therapy, Videoconferencing, Cancer, Pain, Emotional distress, Quality of Life

## Abstract

Pain is a common symptom in patients with cancer accompanied by anxiety, depression, and worsening Quality of Life (QoL). Cognitive-Behavioral Therapy (CBT) has proven to be effective in the management of these symptoms, although access to it faces multiple barriers. While Videoconferencing can be an option, its application in patients with cancer and chronic pain in Latin America remains limited. To evaluate the preliminary feasibility and acceptability of CBT via videoconferencing to improving pain, anxiety, depression, and quality of life in Mexican patients with cancer and chronic pain. This pilot feasibility study used a pre-experimental design with a single group and pre-test and post-test measurements. Patients received a five-session psychological intervention via videoconferencing. The feasibility criteria were: eligibility rate ≥ 50%, enrollment rate ≥ 50%, attendance rate ≥ 70%, & ≥ 80% of the acceptability items ≥ 8. Ten participants were enrolled and agreed to participate completing all 5 sessions (attendance rate = 100%). Participants reported high elegibility rate (71.4%), acceptability of the intervention (99%), comprehensibility (95%) and usefulness (94%). The majority (99%) indicated that they would recommend the psychological intervention to others. Exploratory analyses showed changes in pain improvement (d = .899), anxiety symptoms (d = 1.36), depression symptoms (d = .755), and quality of life (d = .788). CBT via videoconferencing represents a viable and well-accepted alternative for the psychological treatment of patients with cancer and chronic pain in Mexico. This modality could expand access to care when face-to-face intervention is not possible.

## Introduction

Approximately 70% of people with cancer experience pain, and nearly half of these cases are not adequately managed, even after treatment is completed (Pergolizzi et al., 2023). In Mexico, the cancer population reports moderate levels of pain according to the Numerical Rating Scale (Breivik et al., 2008), with a median of 4 points (Mendoza et al., 2025) and an average of 5.6 in patients seen for the first time at the Pain Clinic (Sánchez Ortega et al., 2024).

This symptom often coexists with mental health disorders such as anxiety and depression, which can intensify the perception of pain by increasing attention to it (Bushnell et al., 2015; Cramer et al., 2018; Eaton, 2021; González, 2014; Otis, 2007). In turn, pain interferes with multiple aspects of daily life, negatively affecting patients' Quality of Life (QoL) (Barrett et al., 2017; Decoster et al., 2019; Expósito-Vizcaíno et al., 2020; Mu’taz & Hamdan-Mansour, 2016).

Pharmacological treatment is the first line of treatment for pain management in patients with cancer (Somers et al., 2015). However, due to the complexity of pain and its multidimensional nature—biological, psychological, cognitive, social, and cultural—(Debbağ & Khidhir,^2016^), various institutions such as the National Comprehensive Cancer Network (NCCN), the National Institutes of Health (NIH), and the International Association for the Study and Treatment of Pain (IASP) promote a comprehensive approach that combines pharmacological and non-pharmacological treatments (Miaskowsk & Cleary, 2005; International Association for the Study of Pain, 2023; Somers et al., 2015; Swarm et al., 2025).

Among non-pharmacological interventions, cognitive behavioral therapy (CBT) has been shown to be effective in the treatment of chronic pain and is considered the standard in psychotherapy for this condition (Williams et al., 2021). In addition, the benefits of this intervention have been consistently reported for decreased symptoms of anxiety and depression and increased quality of life in this same population (De Paolis et al., 2019; Kelleher et al., 2019; Mehta et al., 2019; Parás-Bravo et al., 2018; Powers et al., 2017; Ye et al., 2018).

Despite the effectiveness of CBT in pain management and mental health, access to it is often limited by barriers such as cost, transportation to the hospital, a shortage of specialized professionals, and long waiting times for appointments ( Burke, 2019; Dear et al., 2013; Eccleston et al., 2014; Mehta et al., 2019; Wright et al., 2019). In Mexico, these difficulties are exacerbated by logistical problems such as lack of time, limited transportation, or difficulties in scheduling appointments (Benjet et al., 2020). Given this situation, the use of digital health technologies (e-Health) is positioned as a promising alternative to expand access to evidence-based psychological interventions. In particular, CBT via videoconferencing has shown benefits in reducing pain, anxiety, and depression in people with conditions such as headache, chronic low back pain, rheumatoid arthritis, osteoarthritis, and fibromyalgia (Buhrman et al., 2016; Dear et al., 2013; Eccleston et al., 2014; Mehta et al., 2019; Wright et al., 2019). However, the implementation of CBT via videoconferencing has been understudied in the management of chronic pain in cancer patients, and the limited evidence available comes mainly from developed countries (Kelleher et al., 2019; Somers et al., 2016; Winger et al., 2023). These studies may not adequately represent the sociocultural and economic realities of Latin America, which have a significant influence on the approach to pain (Crimmel et al., 2024; IASP, 2022; Ng et al., 2019). Therefore, before proceeding with efficacy evaluations, or considering large-scale implementation in oncology settings, it is essential to generate local evidence to determine the feasibility, acceptability, and clinical relevance of videoconference-based CBT in the Mexican population (Ruda-Santolaria et al., 2023; Somers et al., 2015).

Therefore, the main aim of this study was to evaluate the preliminary feasibility and acceptability of CBT via videoconferencing in a public cancer hospital in Mexico. A secondary objective was to explore preliminary improvements in pain, symptoms of anxiety and depression, and quality of life in patients with cancer and chronic pain.

## Method

A single-group pre-experimental design with pre- and post-intervention measurements was used (Kerlinger & Lee, 2002). As a pilot study aimed at assessing the preliminary feasibility and acceptability of the intervention, no statistical power calculation was performed, in accordance with the recommendations for studies of this type (Althubaiti, 2022; Hertzog, 2008). In pilot studies, the use of small samples is appropriate, as the main objective is to examine operational, implementation, and feasibility aspects rather than to estimate effects accurately (Hertzog, 2008; Leon et al., 2011). In line with standard practice in pilot feasibility studies in people with pain, 10 participants were recruited (Failo et al., 2021; Reneau & Nichols, 2022; Zambelli et al., 2024). Recruitment took place at the INCan Pain Clinic (Mexico) between 9 August 2024 and 6 January 2025.

The inclusion criteria were: 1) cancer diagnosis, 2) active follow-up by the pain clinic, 3) Participants who reported mild to moderate pain intensity, defined as a score of 1 to 6 on the Numeric Rating Scale (NRS) (Pedrajas & Molino, 2008). In addition, participants presented mild to moderate symptoms of anxiety and/or depression, operationalized as scores ranging from 6 to 11 on the corresponding subscales of the Hospital Anxiety and Depression Scale (HADS) (Galindo et al., 2015), 4) ≥ 18 years of age, 5) Karnofsky index ≥ 60, and 6) access to a computer, cell phone, or tablet, internet connection, a private space, and basic knowledge of how to conduct a video conference via Zoom or WhatsApp, or support from a family member who can operate the equipment.

The Exclusion criteria: 1) receiving psychological and/or psychiatric treatment at the time of invitation to participate in the intervention.

## Instruments

### Sociodemographic and Clinical Questionnaire

A questionnaire was designed to collect sociodemographic and clinical data: age, sex, educational level, place of residence, cancer diagnosis and stage, medical treatment, functionality, and pain characteristics (Anatomical area of main pain, affected areas, location, and duration of the main pain).

### Numerical Rating Scale (NRS)

Participants were asked to rate the intensity of their pain at the time of assessment from 0 to 10 (where 0 is no pain and 10 is the worst pain imaginable). (Safikhani et al., 2018).

### Short Form McGill Pain Questionnaire (SF-MPQ)

Developed by Melzack (1987) and validated in Mexico by Mendoza-Contreras et al. (2025), it assesses pain in patients with cancer & chronic pain. It consists of two dimensions: affective-nociceptive (5 items that assess the degree to which pain is perceived as cruel, exhausting or fearful) and neuropathic (4 items that assess pain sensations in areas of the body such as bursting or hurting). It includes a Visual Analog Scale (VAS) ranging from 0 (no pain) to 100 (worst possible pain), and an item that measures current pain intensity from 0 (no pain) to 5 (unbearable). Descriptors are rated on a Likert scale from 0 (no pain) to 3 (severe). It has adequate fit indices (CFI = 0.960, GFI = 0.962, RMSEA = 0.061) and overall internal consistency of α = .82- and ω = .82. The total explained variance was 39.82%.

### Hospital Anxiety and Depression Scale (HADS)

Developed by Zigmond and Snaith (1983) and validated in Mexico by Galindo et al. (2015), it is a self-administered instrument consisting of 12 items: 6 for anxiety (which is defined as a state of worry, tension or panic in everyday situations) and 6 for depression (which focuses on assessing the state of anhedonia, considered a central element of the clinical picture).). Each item is rated from 0 (minimum) to 3 (maximum). It has an overall reliability of α = 0.86 and an explained variance of 48.04%. For depression, α = 0.80 and a variance of 11.49%; for anxiety, α = 0.79 and a variance of 36.55%. The cut-off points are: 0–5 (no symptoms), 6–8 (mild), 9–11 (moderate), and ≥ 12 (severe), for both anxiety and depression.

### Quality of Life Questionnaire of the European Organization for Research and Treatment of Cancer (EORTC QLQ-C30)

Developed by Aaronson et al. (1993) and validated in Mexico by Oñate-Ocaña et al. (2009), it consists of 30 items: 28 with a scale of 1 to 4 and two with a scale of 1 to 7. It assesses three dimensions: functional (physical, role, cognitive, emotional, and social), symptoms (fatigue, pain, nausea, and vomiting), and overall health/quality of life. It has an internal consistency of α = 0.90 and concurrent validity with the Karnofsky scale (p < 0.001).

## Access Variables

### Feasibility

The feasibility of CBT via videoconferencing was assessed using three main measures: the eligibility rate (participants who met the inclusion criteria out of the total assessed), the enrollment rate (participants enrolled out of the total eligible) and the attendance rate (proportion of sessions attended relative to the total planned) (Lim et al., 2017; Price et al., 2017). Additionally, other variables related to the implementation process were recorded: study dropout, adherence to assigned tasks—recorded weekly on a scale of 0 to 3—and the total duration of the intervention (number of weeks between session 1 and session 5) (Babiano-Espinosa et al., 2019; Rodin et al., 2020). The tasks included: in session 2, recording dysfunctional thoughts related to pain, anxiety, and depression, as well as daily deep breathing practice; in session 3, recording cognitive restructuring; and in session 4, daily recording of progressive muscle relaxation and guided imagery practice.

### Acceptability

A checklist designed for this study was used to assess the acceptability of CBT via videoconferencing in patients with cancer and chronic pain. To develop it, the literature was reviewed and the seven components proposed by Sekhon et al. (2017) were used: affective attitude, burden, opportunity cost, perceived efficacy, self-efficacy, consistency of the intervention, and intention to adhere.

The list was structured into three sections: 1) acceptability of CBT via videoconferencing (10 items), 2) relevance of the intervention and therapeutic techniques (6 items), and 3) additional observations (5 items). The responses to the 10 items in the first section were rated using an 11-point Likert scale (0–10), with higher scores reflecting greater acceptability. The section included six items worded in a positive sense and four in a negative sense.

It was constructed based on the Theoretical Framework of Acceptability (Sekhon et al., 2017), the Treatment Acceptability/Adherence Scale (Milosevic et al., 2015), the Client Satisfaction Questionnaire (Attkisson & Zwick, 1982), and other pilot studies (Nguyen et al., 2018; Reneau & Nichols, 2022). It was reviewed by four expert psychologist specializing in psycho-oncologys, obtaining an overall Aiken *V* > .70, which demonstrated adequate content validity.

## Procedure

Participants were recruited at the INCan Pain Clinic in Mexico. The principal investigator conducted recruitment after medical consultations with algologists, who informed patients about the protocol as part of a comprehensive approach to pain management. Subsequently, the initial assessment was carried out in the psychology office, the objectives and procedures of the study were explained, and patients who agreed to participate signed the informed consent form.

Those who met the eligibility criteria underwent the baseline assessment and received the supplementary material. Finally, their contact details were recorded to coordinate the following sessions, which were conducted via videoconference.

### *Cognitive Behavioral Therapy *via* Videoconferencing*

The intervention was delivered by a psychologist specializing in psycho-oncology (first author) with formal training in CBT and comprehensive pain management and seven years of experience in the field. It was structured in five sessions, each lasting between 60 and 150 min. The initial assessment was conducted in person, after the patient's consultation at the Pain Clinic, while the remaining sessions (including the final assessment) were conducted via videoconference (Zoom or WhatsApp), with participants located in their respective homes.

The intervention was organized based on an ongoing systematic review protocol registered on the PROSPERO platform (Mendoza-Contreras et al., 2024), complemented by the Managing Chronic Pain manual (Otis, 2007) and an intervention protocol focused on training pain coping skills in patients with cancer, based on mobile care (Somers 2015, 2016).

It included psychoeducation techniques, cognitive restructuring (thought change), diaphragmatic breathing, progressive muscle relaxation, and guided imagery. The objectives were: a) to modify dysfunctional thoughts related to pain, anxiety, and depression, b) to regulate both hypervigilance toward bodily sensations and the exacerbated physiological response to pain and anxiety. The clinical goals were to reduce pain and emotional symptoms and improve quality of life.

The intervention was based on the cognitive-behavioural model (CBT) of chronic pain management, which posits that the experience of pain is modulated by cognitive, emotional, and behavioural processes, in addition to nociceptive stimuli (Bose, 2024; Driscoll et al., 2021; McCracken & Vowles, 2014) within a biopsychosocial approach (Flor et al., 2023). The components of each session are briefly described below:

### Session 1

Presentation of objectives, clinical interview and initial assessment. Support materials (manual, audio and relaxation videos) were provided to promote self-management and adherence (Addis et al., 1997; Otis, 2021).

### Session 2

The impact of pain on quality of life was addressed and the cognitive-behavioural model applied to pain was introduced, highlighting how thoughts and emotions (anxiety and depression) modulate pain perception. Diaphragmatic breathing was taught to promote physiological and emotional self-regulation, helping to reduce perceived intensity and improve the feeling of control (McCracken & Vowles, 2014; Vambheim et al., 2021).

### Session 3

Changing dysfunctional thoughts related to pain, anxiety, or depression, based on Beck's cognitive model. This seeks to reduce catastrophising and promote more effective coping, decreasing pain intensity and interference (Beck, 2020; Petrini & Arendt-Nielsen, 2020).

### Session 4

Training in progressive muscle relaxation and guided imagery, physiological regulation techniques of CBT. These strategies decrease sympathetic activation and promote calmness and control, improving perception of pain and emotional distress (McCracken & Vowles, 2014; Porges, 2011; Vambheim et al., 2021).

### Session 5

The contents of the previous sessions were reviewed, questions were answered, and the techniques learned were reinforced. Finally, the post-intervention assessment and acceptability checklist were administered.

Throughout the intervention, the techniques were taught, practiced, and reviewed in session. Homework assignments were given, and participants were asked to record their progress in the support manual.

## Statistical Analysis

The data were analyzed using IBM SPSS version 26. Descriptive statistics were calculated for sociodemographic, clinical, and study variables. Preliminary feasibility and acceptability were the primary outcomes. Feasibility was defined a priori using parameters commonly used in pilot trials: Eligibility rate ≥ 50% (Rocque et al., 2017), enrollment rate ≥ 50% (Tetmeyer et al., 2025), Attendance rate ≥ 70% (Azizoddin et al., 2025); and Acceptability if at least 80% of the 10 items in the first section of the acceptability checklist are rated ≥ 8 (Azizoddin et al., 2025).

As secondary/exploratory outcomes, pre- and post-intervention changes in pain, anxiety and depression symptoms, and participants' quality of life were assessed.

Although the study did not have the statistical power necessary to evaluate efficacy, exploratory analyses were performed to estimate the effect size using Cohen's d, along with its 95% confidence interval (95% CI) using the escalc function of the metafor library in R. The values were interpreted according to Cohen's criteria (1988): 0.2 (small), 0.5 (medium), and ≥ 0.8 (large).

## Ethical Considerations

This research protocol was approved by the Research Ethics Committee and the Research Committee of INCan (Mexico) under approval number (*XXX/XXX/XXX*) (*XXX/XXX/XX: MASKED FOR ANONYMOUS REVIEW*). The study was conducted in accordance with the standards of the Declaration of Helsinki, and each participant agreed to participate by voluntarily signing an informed consent form.

## Results

**Fig. 1:**
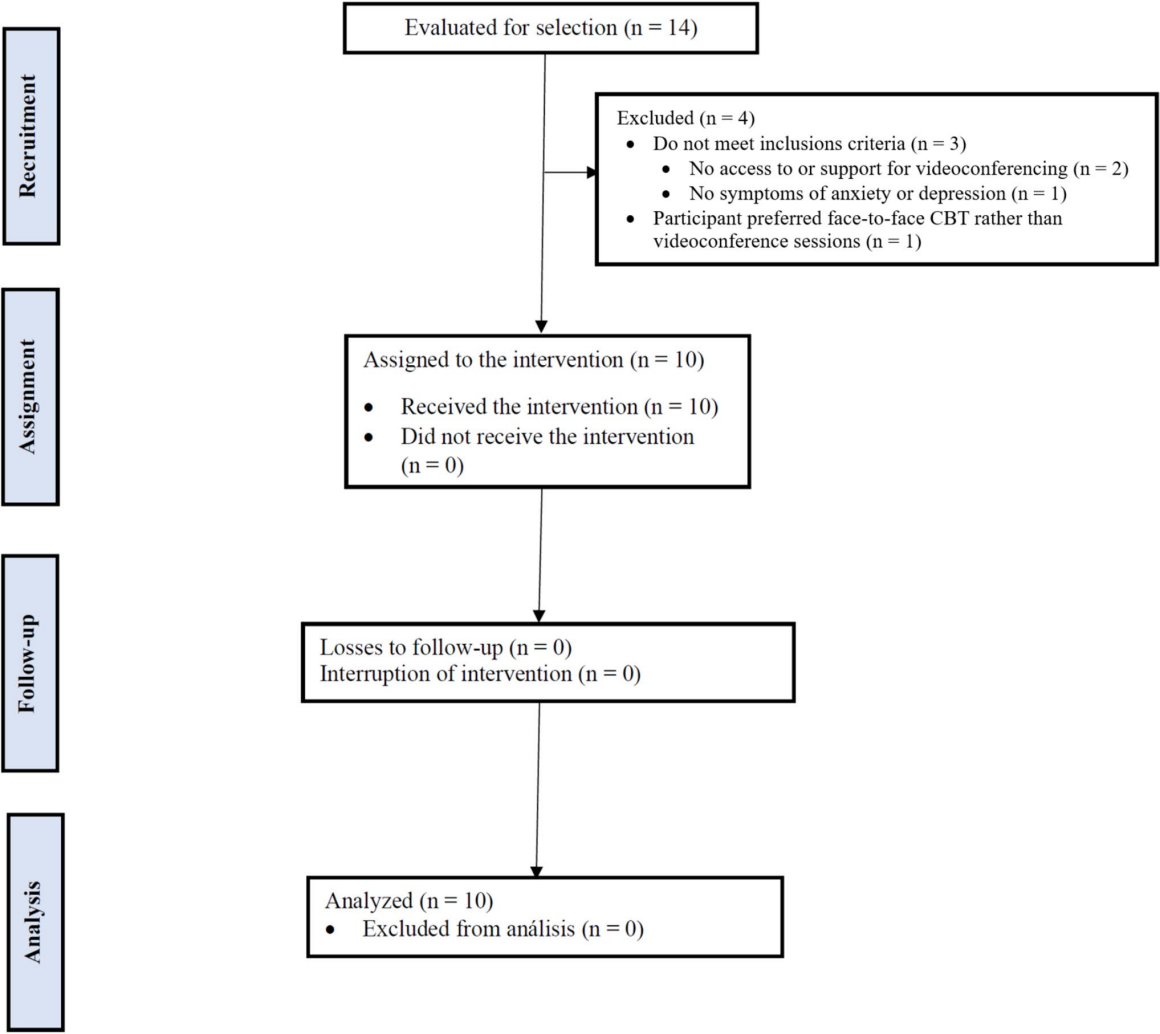
Flow chart through the phases of the study. Adapted from the CONSORT diagram (Cobos-Carbo & Augustovski, 2011)

Fourteen potential participants were considered for the intervention, of whom 10 met the inclusion criteria and agreed to participate. The reasons for exclusion are shown in Fig. 1.

Table 1 shows the sociodemographic and clinical characteristics of the participants. The average age was 46.1 years (SD = 14.0; range = 24–63). Most were women (70%), with upper secondary education (70%), married (50%), self-employed (40%), and residents of the State of Mexico (60%). In terms of clinical history, 60% had received previous psychological care. Thirty percent had been diagnosed with breast cancer, 33.3% were in stage IV, and 60% had comorbidities. Eighty percent were undergoing active cancer treatment (hormone therapy being the most common). Most reported having high social support at the time of the pre-evaluation.

**Table 1:** Sociodemographic and clinical characteristics of participants. *Source*: Own elaboration

Age (Years) x̅ = 46.10 SD = 14.00 Range (24–63)					
	n	%		n	%
Gender			Type of cancer by location/system		
Women	7	70.0	Breast	3	30.0
Men	3	30.0	Musculoskeletal	2	20.0
Academic grade			Genitourinary	2	20.0
Elementary	1	10.0	Hematological/blood	1	10.0
Middle school	7	70.0	Digestive/gastrointestinal	1	10.0
High school	2	2.0	Gynecological	1	10.0
Marital status			Clinical Stage^a^		
Single	4	40.0	In situ	1	16.6
Married	5	50.0	I	1	16.6
Widowed	1	10.0	II	1	16.6
Occupation			III	1	16.6
Household	3	30.0	IV	2	33.3
Self-employed	4	40.0	Comorbidity		
Unemployed	2	20.0	Yes	6	60.0
Student	1	10.0	No	4	40.0
Residence			Number of comorbidities^a^		
Mexico City	1	10.0	One	2	33.3
State of Mexico	6	60.0	Two	2	33.3
Interior of the republic	3	30.0	Three	2	33.3
Social support			Undergoing cancer treatment		
Low	0	0.0	Yes	8	80.0
Moderate	3	30.0	No	2	20.0
High	7	70.0	Type of treatment		
Mental health service			Surgery	1	12.5
Yes	6	60.0	Chemotherapy	2	25.0
No	4	40.0	Hormone therapy	3	37.5
Type of mental health service			Targeted therapy	1	12.5
Psychology	6	100.0	Surgery + chemotherapy + targeted therapy	1	12.5
Psychiatry	0	0.0			

^a^ = 6 participants; ^b^ = 6 participants; x̅ = mean; SD = Standard; n = Participants

The duration of the main pain was Md = 10 months. Most participants (70%) reported experiencing continuous pain, and the most frequent algological diagnosis was post-surgical pain (28.5%) (see Table 2).

**Table 2:** Pain characteristics

Numerical Rating Scale: Md = 4.5 Time with main pain: Md = 6 months (Range = 1–36)					
	n	%		n	%
Anatomical area of main pain			Diagnosis of cancer-related pain		
Head and neck	2	20.0	Yes	7	70.0
Upper extremities	1	10.0	No	2	20.0
Lower extremities	4	40.0	Not specified	1	10.0
Abdomen	2	20.0	Type of cancer-related pain^a^		
Gluteus	1	10.0	Bone pain	1	14.2
Temporality			Neuropathic pain	1	14.2
Continuous	7	70.0	Post-surgical pain	2	28.5
Intermittent	3	30.0	Pain caused by medication	1	14.2
Current pharmacological treatment			Two or more types of pain	2	28.5
Non-opioid analgesics	4	40.0	Interventional treatment in the past		
Weak opioids	2	20.0			
Strong opioids	4	40.0	Yes	4	40.0
Adjuvants	7	70.0	No	6	60.0
Anatomical areas with different pains reported at the time of evaluation			Type of treatment		
Two	3	30.0	Nerve block	3	75.0
Three	1	10.0	Prolotherapy	1	25.0
Four or more	6	60.0			

^a^ = 7 participants; Md = Median; n = Participants

## Feasibility

Of the 14 participants evaluated, 4 did not meet the inclusion criteria, representing an eligibility rate of 71.4%. The main reasons for ineligibility were lack of access to an electronic device, internet connection, or basic knowledge to conduct videoconferences via Zoom or WhatsApp, or lack of family support to operate the equipment (n = 2). In addition, one participant was excluded for not presenting symptoms of anxiety and depression (n = 1) and another for preferring a face-to-face intervention (n = 1). All eligible participants agreed to participate, resulting in a 100% enrollment rate.

All participants (100%) completed the five sessions; 40% did so within the estimated time (4 weeks) and 40% completed the three weekly activity logs.

During the videoconference sessions, some technical and environmental issues arose, all of which were resolved without interrupting participation: problems with Zoom, resolved using WhatsApp (n = 4); interruptions due to the home environment and lack of privacy (n = 2); brief departures due to family or space issues (n = 1); and power outages/connection failures (n = 1). Ninety per cent of participants connected to the sessions using a mobile phone.

## Acceptability

At the end of the intervention, an acceptability checklist divided into three sections was applied: (1) acceptability of the intervention, (2) relevance of therapeutic techniques, and (3) open-ended comments. Ninety per cent of the acceptability items were rated ≥ 8, and the quality of the program was rated as excellent (99%). A more detailed description of the acceptability items is shown in Table 3. The techniques considered most useful and applicable in everyday life for managing pain and emotional distress were in order: 1) cognitive restructuring, 2) psychoeducation about pain and diaphragmatic breathing, 3) guided imagery, and 4) progressive muscle relaxation.

**Table 3:** Acceptability assessment of cognitive-behavioural intervention via videoconferencing

Item	Mean (0–10)	*DE*	%
1. Las técnicas psicológicas que recibí me fueron útiles [1. The psychological techniques I received were useful to me.]	9.40	.84	94.0
2. Estoy satisfecho con la terapia psicológica por videoconferencia [2. I am satisfied with psychological therapy via videoconference.]	9.80	.63	98.0
3. Esta terapia psicológica por videoconferencia me pareció cansada [3. I found this psychological therapy via videoconference tiring.]	9.70	.67	97.0
4. Me gustó participar en esta terapia psicológica por videoconferencia [4. I enjoyed participating in this psychological therapy via videoconference.]	9.70	.67	97.0
5. Esta terapia psicológica por videoconferencia tuvo efectos negativos (por ejemplo, mareo o cansancio) [5. This psychological therapy via videoconference had negative effects (e.g., dizziness or fatigue).]	10.00	.00	100
6. Esta terapia psicológica por videoconferencia me ayudó a manejar mi dolor y malestar emocional [6. This psychological therapy via videoconferencing helped me manage my pain and emotional distress.]	9.70	.67	97.0
7. Preferiría tomar terapia psicológica presencial en lugar de videoconferencia [7. I would prefer to have psychological therapy in person rather than via videoconferencing.]	5.40	3.56	54.0
8. Preferiría recibir solo medicamento para mi dolor en lugar de ambos tratamientos (medicamento y terapia psicológica) [8. I would prefer to receive only medication for my pain rather than both treatments (medication and psychological therapy).]	9.00	3.16	90.0
9. Las técnicas proporcionadas durante la terapia psicológica por videoconferencia fueron fáciles de comprender [9. The techniques provided during psychological therapy via videoconference were easy to understand.]	9.50	.85	95.0
10. Recomendaría estar terapia psicológica por videoconferencia a un amigo o conocido con cáncer y dolor crónico [10. I would recommend psychological therapy via videoconference to a friend or acquaintance with cancer and chronic pain.]	9.90	.31	99.0

Note: The table includes the means and standard deviations of the items. To facilitate interpretation, the means were converted to percentages. Items 3, 5, 7, and 8 were reversed due to their directionality

Participants reported a perceived decrease of 80% in primary pain (SD = 9.2), 88% in anxiety (SD = 9.2), 86.5% in depression (SD = 11.1), and an 88.6% improvement in quality of life (n = 7). In addition, they reported an 80.5% reduction (SD = 15.7) in all referred pain during the assessment.

## Open Comments on Feasibility and Acceptability

Although no formal qualitative interview was conducted, open comments from the checklist complemented the quantitative findings. Participants highlighted the convenience, flexibility, and time savings offered by the online modality:

- ‘*I liked the video call, it's convenient from home, the flexibility of time, and avoiding the trip to the hospital*’ (Male, 63 years old, prostate cancer).
- ‘*I like both ways (face-to-face therapy or videoconferencing), but videoconferencing is practical and I'm in a comfortable place*’ (Male, 29 years old, testicular cancer).
- ‘*I prefer both methods (face-to-face therapy or videoconferencing), but it's difficult for me to come to the hospital because of the time, distance and money involved. During videoconference therapy, I'm at home and don't have to travel*.’ (Woman, 58 years old, breast cancer)

Regarding the usefulness of the intervention, participants highlighted the application of techniques for managing pain and regulating emotions:

- ‘*Changing my thoughts to something more positive and putting a stop to the negative ones further improves my pain and everything around me*’ (Woman, 58 years old, breast cancer).
- ‘*Deep breathing, muscle relaxation, and guided imagery have helped me a lot to lower my pain levels*.’ (Woman, 50 years old, sarcoma).
- ‘*I felt helped, listened to, and understood in terms of my pain*.’ (Woman, 24 years old, leukaemia).

## Exploratory Results of the Intervention

Table 4 presents the pre- and post-treatment scores (X̄, SD), together with the effect sizes (Cohen's d). After the intervention, improvements were observed in overall pain ( d = 0.899 [95% CI = -0.02, 1.81]), in the affective-nociceptive dimension (*d* = 1.06 [95% CI = .126, 1.99]), in the neuropathic dimension (*d* = .455 [95% CI = .432, 1.34]), anxiety symptoms (*d* = 1.36 [95% CI = .388, 2.33]), and depressive symptoms (*d* = 0.755 [95% CI = -0.151, 1.66]). Similarly, an improvement in overall quality of life was found (*d* = 0.788 [95% CI = 1.69, –0.121]).

**Table 4:** Effect size in pain, anxiety, depression, and overall quality of life

Variable	Pre–test		Post–test				
	x̄	SD	x̄	SD	d	95% CI	Interpretation
McGill total	7.60	4.81	3.40	4.11	.899	–.020, 1.81	Large effect
Affective–nociceptive dimension	4.70	3.12	1.70	2.21	1.06	.126, 1.99	Large effect
Neuropathic dimension	2.90	2.80	1.70	2.21	.455	–.432, 1.34	Medium effect
VAS	47.95	21.28	35.15	22.74	.558	–.334, 1.45	Medium effect
PPI	1.30	0.82	1.00	0.94	.430	–.456, 1.31	Small effect
HADS anxiety	7.90	3.47	3.10	3.28	1.36	.388, 2.33	Large effect
HADS depression	6.00	3.12	3.20	3.93	.755	–.151, 1.66	Medium effect
EORTC total	60.83	13.05	72.50	15.23	.788	1.69, –.121	Large effect

x̄ = Mean; SD = Standard Deviation; VAS = Visual Analog Scale; PPI = Current pain intensity indicator

## Discussion

Pain in patients with cancer is a complex experience that requires a comprehensive, multidimensional, and multidisciplinary approach. The primary objective of this study was to evaluate the preliminary feasibility and acceptability of CBT via videoconferencing in a public cancer hospital in Mexico. The secondary objective was to explore trends in improvement in pain, symptoms of anxiety and depression, and quality of life. To our knowledge, this is the first study in Latin America to apply this type of intervention in patients with cancer and chronic pain, showing promising results that support its potential as a therapeutic complement.

An eligibility rate of 71.4% and an enrollment and attendance rate of 100% were observed, exceeding the pre-established feasibility thresholds (Azizoddin et al., 2025; Rocque et al., 2017; Somers et al., 2015; Tetmeyer et al., 2025). These results are closely related to the recruitment process carried out in the  Pain Clinic, where medical staff suggested psychological care as part of comprehensive pain management. The doctor-patient relationship, recognised for its influence on treatment acceptance (Licciardone et al., 2024), probably contributed to increasing patients' willingness to participate. From the perspective of Garret et al.“s (2021) model of barriers and facilitators, medical recommendation increases patients” familiarity with non-pharmacological interventions, reinforcing their perception of usefulness and legitimacy and, consequently, their willingness to use them.

It is important to note that all participants who began the intervention attended the five scheduled sessions and completed the post-intervention assessment, a remarkable finding even considering the small sample size. However, it is necessary to continue evaluating these indicators in future studies, especially in the Mexican context, where financial compensation for participating in clinical studies or pilot studies such as ours is not common due to funding limitations, unlike what has been reported in feasibility studies conducted in developed countries, in which participants received financial incentives (Azizoddin et al., 2025; Reneau et al., 2022).

Regarding technological feasibility, most participants accessed the study via Zoom; however, in some cases, it was necessary to resort to WhatsApp video calls due to unfamiliarity with the platform, as well as interruptions due to the home environment, lack of privacy, and connection failures. These situations are to be expected in feasibility studies, whose purpose is to identify logistical barriers and necessary refinements before implementing more robust studies (Eldridge et al., 2016; Lancaster, 2015). These observations provide valuable information for adapting the intervention to the Mexican context and improving its future implementation.

The fact that 90% of participants connected from a mobile phone highlights the importance of designing interventions that are compatible with mobile devices and considering digital accessibility as a central element (Mayhew et al., 2023; Somers et al., 2015). This is especially relevant in contexts where pain care services are centralised in tertiary hospitals and are not easily accessible outside urban areas, as is the case in Mexico and Latin America (Lincoln et al., 2022; Pergolizzi et al., 2019). Given that mobile Internet use is increasingly common in middle-income countries (Benjet et al., 2020), remote interventions represent an opportunity to reduce these structural barriers, provided that technological difficulties are anticipated and connection alternatives are offered to facilitate their implementation.

The intervention showed high acceptability. Ninety per cent of items scored ≥ 8, exceeding what was reported in previous studies (Azizoddin et al., 2025), and 99 per cent of participants rated the intervention as excellent. Only 20% expressed a preference for face-to-face sessions, although they noted that factors such as cost, time, and distance would have made it difficult for them to attend. This highlights the value of videoconferencing in expanding access, especially considering that 90% of participants lived outside the urban area near the hospital and 60% had at least one comorbidity. In this context, a study conducted in the German population reported that CBT via videoconferencing is not only effective but also more economical than face-to-face sessions, with a 76% probability of being cost-effective, as it offers good results with a lower investment (Baumann et al., 2020).

Furthermore, 99% of participants indicated that they would recommend the intervention to a family member or acquaintance with cancer and chronic pain, reinforcing satisfaction with the intervention and suggesting potential for promoting support networks among patients who are often unaware of the role of non-pharmacological approaches in pain management, particularly psychology and CBT (Garret et al. 2021).

Furthermore, exploratory results showed trends of improvement in pain, anxiety, depression, and quality of life, with preliminary effects of medium and large magnitude. These changes are consistent with evidence that CBT can modify cognitive and emotional processes involved in the experience of pain (Flor et al., 2023; Mercer Lindsay et al., 2021) and reduce symptoms of anxiety and depression (Liu et al., 2022; Ye et al., 2018), which directly influence pain perception (Bushnell et al., 2015; Cramer et al., 2018). Although these findings should be interpreted with caution due to the sample size and study design, they are consistent with previous studies of CBT in cancer patients with chronic pain (Somers et al., 2015, 2016; Bernier et al., 2020) and provide preliminary evidence of its potential to improve physical and emotional dimensions related to quality of life.

Based on these preliminary findings of feasibility and acceptability, future research should have a larger sample size and incorporate a semi-structured interview to delve deeper into participants' experiences and identify additional barriers and facilitators. It will also be important to explore improvements to the intervention, such as the inclusion of behavioural components (e.g., problem solving and behavioural activation) and monitoring their effects over time. In addition, it will be important to evaluate other variables, such as pain catastrophising, to better understand the mechanisms of change. Finally, the technological incidents observed underscore the need to strengthen support in the use of digital platforms and consider alternative connections to optimise their implementation in subsequent studies.

Among the main limitations of this study are the small sample size and the lack of a control group, which restricts the generalisation of findings on feasibility and acceptability. Although all participants who started the intervention completed the sessions and the post-intervention assessment, the limited number of participants reduces the ability to identify additional barriers and facilitators. Furthermore, a qualitative component, such as semi-structured interviews, was not included, which would have allowed for a deeper understanding of participants' perceptions of the acceptability of the intervention.

Another relevant limitation is technological accessibility: of the 14 potential participants, 2 did not have the necessary technology, knowledge, or Internet access to participate, suggesting that a percentage of the population may not be able to benefit from this type of remote intervention. This highlights the need for digital support strategies and alternative connection options in future implementations, especially considering that, although the majority of the Mexican population has Internet access (100.2 million, 83.1% of the population aged 6 years and older, with 95.1% from home; INEGI, 2025), training in the use of e-health services can vary.

Finally, as this is a single-group pilot feasibility study, the findings should be interpreted as preliminary and descriptive, focusing on feasibility rates and reported acceptability (Teresi et al., 2022). According to Teresi et al. (2022), it is recommended to report effect sizes accompanied by their confidence intervals, as was done in this study. In this case, all variables whose 95% CI crosses zero should be interpreted with caution, despite having obtained moderate or large effects.

## Conclusions

CBT via videoconferencing for Mexican patients with cancer and chronic pain proved feasible and well accepted in a public cancer hospital, with promising preliminary results. Recruitment in the  Pain Clínic, where psychological care was presented as part of comprehensive treatment, facilitated participation, and the checklist data together with open-ended comments reflected high acceptability. Some technical and environmental issues were identified during the sessions and should be considered in further research. Despite the small sample size, preliminary medium- and large-effect sizes were observed in pain, anxiety, depression, and quality of life. These findings suggest that CBT via videoconferencing could be a promising therapeutic alternative; however, further research is needed with larger and more methodologically rigorous studies to further investigate the feasibility and acceptability and identify the statistical effects of the intervention in the Mexican oncology context.

## Data Availability

The data that support the findings of this study are available from the correspondence author upon reasonable request and with the permission of Instituto Nacional de Cancerología (INCAN) from Mexico due to participants privacy.
